# A fully defined static suspension culture system for large-scale human embryonic stem cell production

**DOI:** 10.1038/s41419-018-0863-8

**Published:** 2018-08-30

**Authors:** Xia Li, Ruoyu Ma, Qi Gu, Lingmin Liang, Lei Wang, Ying Zhang, Xianning Wang, Xin Liu, Zhongwen Li, Jinhui Fang, Jun Wu, Yukai Wang, Wei Li, Baoyang Hu, Liu Wang, Qi Zhou, Jie Hao

**Affiliations:** 1 State Key Laboratory of Stem Cell and Reproductive Biology, Institute of Zoology, Chinese Academy of Sciences, Beijing, 100101 China; 2 University of Chinese Academy of Sciences, Beijing, 100049 China; 3 Beijing Stem Cell Bank, Chinese Academy of Sciences, Beijing, 100190 China; 4 State Key Laboratory of Membrane Biology, Institute of Zoology, Chinese Academy of Sciences, Beijing, 100101 China

## Abstract

Human embryonic stem cells (hESCs) play an important role in regenerative medicine due to their potential to differentiate into various functional cells. However, the conventional adherent culture system poses challenges to mass production of high-quality hESCs. Though scientists have made many attempts to establish a robust and economical hESC suspension culture system, there are existing limitations, including suboptimal passage methods and shear force caused by dynamic stirring. Here, we report on an efficient large-scale culture system, which enables long-term, GMP grade, single-cell inoculation, and serial expansion of hESCs with a yield of about 1.5 × 10^9^ cells per 1.5-L culture, while maintaining good pluripotency. The suspension culture system was enlarged gradually from a 100-mm dish to a 1.8-L culture bag with methylcellulose involvement to avoid sphere fusion. Under the optimal experimental protocol, this 3D system resolves current problems that limit mass production and clinical application of hESCs, and thus can be used in commercial-level hESC production for cell therapy and pharmaceutics screening in the future.

## Introduction

Human embryonic stem cells (hESCs), one among the pluripotent stem cells, can be induced into various types of functional cells under a certain condition in vitro, and play an important role in regenerative medicine^1^. hESC isolation and expansion have been widely reported since the first hESC line establishment in 1998^2–5^. In most previous reports, hESCs were expanded in adherent culture systems supported with feeder cells or matrices^6,7^. A large number of high-quality hESCs, as well as their derivates, are needed for cell therapy. It must be mentioned that about 10^9^–10^10^ functional cells per patient are required to recover the function for solid organs such as the liver, kidney, pancreas, and heart^8,9^. However, conventional two-dimensional (2D) adherent cultures occupy a large space to scale up hESC production^10^. Meanwhile, functional cells derived from 2D differentiation systems have shown the lack of maturity and functional defects by which the conditions supplied are different from the three-dimensional (3D) originals^11^. Consequently, 2D culture platform is not suitable for large-scale expansion and standard production of hESC, while 3D suspension culture systems for expansion and differentiation bring hope for cell therapy^10,12,13^.

At present, several suspension culture methods have been established, such as cell aggregates^14^, microcarriers carrying cells,^15^ and microcapsules with cells embedded in^16^. Two-fold to four-fold higher hESC densities are achieved on matrigel-coated microcarriers than those in 2D cultures^17^. Afterwards, human pluripotent stem cells (hPSCs) are cultured with single-cell inoculation in spinner flasks for more than 10 passages to maintain pluripotency^18^. Another strategy is that of passage in a mechanical way and supplementing functional polymers to the suspension system, which produced a yield of up to 1.4 × 10^8^ hPSCs in a 200-mL cell culture bag^19^. Although some progress has been made in hESC suspension culture, mass production of good manufacturing practices (GMP)-grade hESCs for clinical application remains challenging because of clump formation in static culture systems, shear force damage in dynamic bioreactors, and the low viability caused by suboptimal passage methods^19–21^.

Here, based on the clinical-grade hESC lines our lab derived^22^, we provide a simple, economical, and robust static suspension culture system for scaling up GMP-grade hESC production. By utilizing ultra-low attachement dish, which have low attachment for cells^23^, we obtained optimized seeding density and culture medium, established a 3D culture system with single-cell hESCs for initial seeding, and produced cells in aggregates for proliferation. Then we progressively scaled up the system to cell culture bags while employing methylcellulose to prevent cell conglobation^19,24^, and finally reached a yield of 1.5 × 10^9^ cells per 1.5-L culture system. Importantly, hESCs maintained normal morphology and pluripotency for more than 30 passages in the 3D culture system. In addition, 3D-hESCs have the same differentiation ability as 2D-hESCs during mesenchymal differentiation. Moreover, the system provides great possibility for hESC production in future clinical cell therapy.

## Results

### Establishment of 3D-hESC suspension culture system in ultra-low dish

**Fig. 1:**
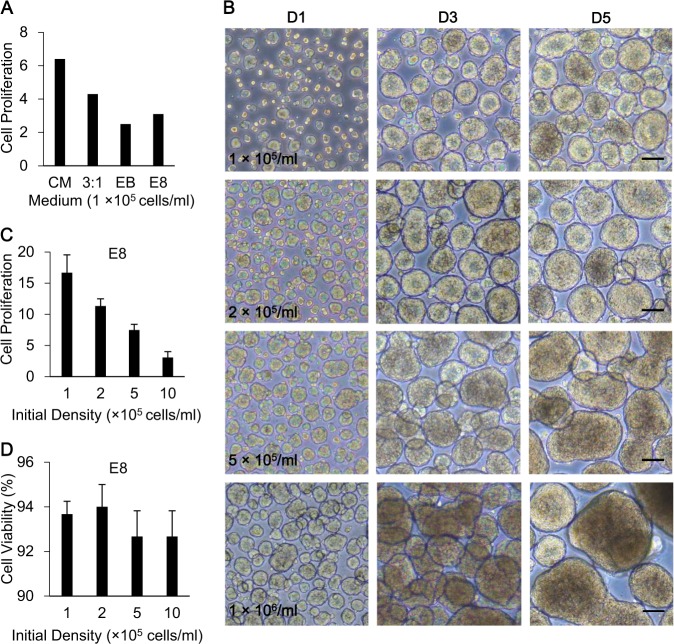
Optimization of 3D human embryonic stem cell (hESC) suspension culture system. **a** Comparison of the average folds of hESC proliferation suspended in different medium. **b** Morphology of hESC spheres with different initial cell seeding density. Scale bar, 100 μm. **c** Comparison of the average folds of hESC proliferation suspended in E8 medium with different initial cell seeding density. **d** Cell viability of spheres suspended in E8 medium with different initial cell seeding density

To establish the massive 3D-hESC culture system, we first optimized the cultivation conditions using a small amount of hESCs in the ultra-low attachment dish. We compared the cell proliferation of hESC spheres suspended in different medium types, including conditioned medium (CM)^25,26^, a suspension culture medium for monkey embryonic stem cells (3:1)^27^, conventional culture medium without bFGF (EB), and Essential 8^TM^ (E8) medium^28^ (Fig. 1a). Considering that CM and 3:1 culture medium both contain fetal bovine serum (FBS), an animal-origin component, which was not recommended for clinical hESC culture^29^, E8 medium was chosen, a fully defined culture medium for hESC suspension culture. We tried to figure out the most suitable cell seeding density for hESC expansion after the comparison of four gradients, by observing sphere morphologies under the microscope during the culture (Fig. 1b). Obviously, the spheres in the groups with an initial density of 2 × 10^5^ cells/ml exhibited more homogeneity, while others with higher seeding densities tended to form big clumps and their spheres were darker in the center on D5 post culture (Fig. 1b). Next, we detected cell proliferation and cell viability by counting cell numbers and trypan staining, respectively, for each seeding density group on D5 post cell culture (Fig. 1c, d), and found that cell proliferation rate declined with the increase of initial density (Fig. 1c). Cell viability was >90% in different seeding density groups (Fig. 1d). Therefore, the density of 2 × 10^5^ cells/ml was chosen for the following experiments.

### Scaling up the hESC sphere culture system to a 1.8-L cell culture bag

**Fig. 2:**
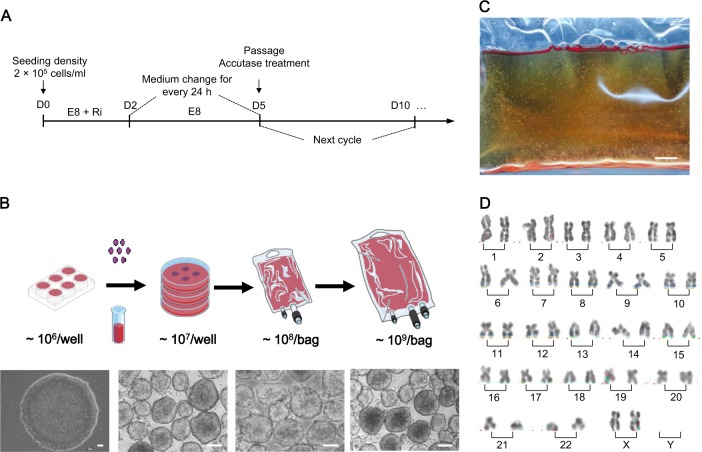
Scaling up human embryonic stem cell (hESC) suspension culture system. **a** Schematic illustration of the subculture cycle. **b** Morphology of 2D cultured hESC colonies and hESC spheres on D5 suspended in 10 cm dish, 200-ml TaKaRa, and 1.8-L TaKaRa. Scale bar, 200 μm. **c** Suspension culture bag comprising hESC spheres. Scale bar, 1 cm. **d** Karyotype analysis of hESC spheres after 10 passages of suspension culture

We next investigated the effects of large-scale culture by suspending hESCs in a 1.8-L cell culture bag with 1.5-L medium (Fig. 2a–c). Large size of hESC colonies would induce differentiation^30^ and the addition of 1% methylcellulose in the culture medium can prevent clump formation^19^. The number of hESCs collected from a 1.5-L cell suspension medium reached 1.5 × 10^9^ on D5 post culture, and spheres were maintained in good shape and condition, which was similar to the ones cultured with small scale (Fig. 2b). The bright figure revealed persistent homogenous sphere distribution throughout the bag (Fig. 2c). Taken together, these data suggested that our 3D culture system could be scaled up to a volume of 1.5-L, which generated a large number of hESCs with high cell viability in the form of cell aggregates with good microscopic morphology.

**Fig. 3:**
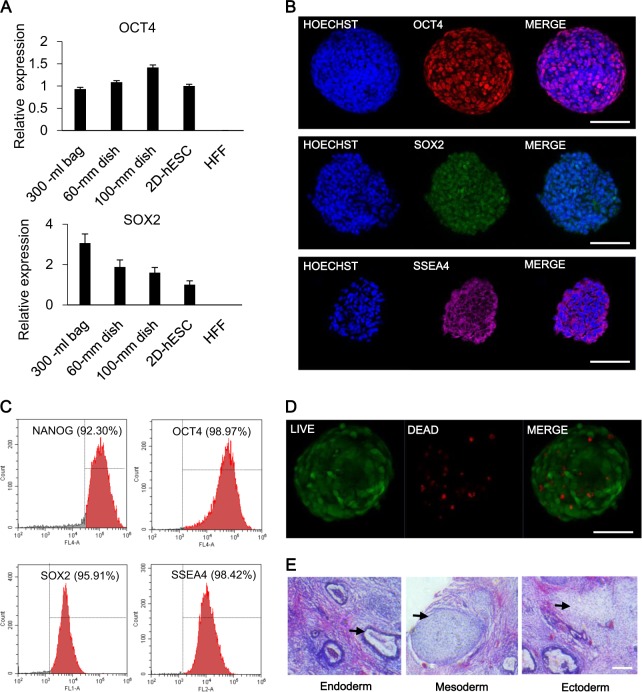
Characterization of human embryonic stem cells (hESCs) in suspension culture **a** Comparative gene expression in hESCs between 2D and 3D culture. Relative gene expression represents data normalized to *GADPH* and expressed relative to 2D-hESC. **b** Immunostaining of hESC spheres with pluripotency markers OCT4, SOX2, and SSEA; the nuclei was stained with DAPI. Scale bar, 100 μm. **c** Flow cytometry of hESCs extracted from spheres and expressing NANOG, OCT4, SOX2, and SSEA4. **d** Dead/live staining revealed cell viability on D5. Scale bar, 100 μm. **e** hESCs cultured in suspension culture produce full teratomas, which contained differentiated cells in all three germ layers, in SCID mice. Arrow: respiratory epithelium (endoderm), cartilage (mesoderm), and brain tissue (ectoderm). Scale bar, 100 μm

Long-term stability is critical for the production of clinical-grade hESCs. During the long-term subculturing (over 30 passages), spheres maintained a normal morphology, with an average diameter of about 208 μm on culture D5 (Supplementary Fig. 1A). The growth curve showed a typical S type^31^, and cell number increased 15-folds at the end of the subculturing (Supplementary Fig. 1B). After being transferred back into 2D culture system, single 3D-hESCs formed colonies with normal morphology (Supplementary Fig. 2). Furthermore, the hESC spheres exhibited normal karyotypes (Fig. 2d) and no abnormal expression in oncogenes. The expression level of *MYC* between 3D-hESCs and Hela showed significant difference^32^, while the level of *p53* between 3D-hESCs and Hela showed no significant difference (Supplementary Fig. 3), which is the same as previously reported by others^33,34^. RT-qPCR results confirmed comparable expression of pluripotent maker, OCT4, and upregulation of SOX2 in 3D spheres compared to 2D adherent colonies (Fig. 3a), which is consistent with previous reports^35,36^. In addition, immunostaining with confocal microscopy revealed cells within spheres; expression of sections showed that all of the cells in a sphere expressed pluripotency markers, OCT3/4, SOX2, and SSEA4 (Fig. 3b). Ubiquitous expression of pluripotent markers NANOG, OCT4, SOX2, and SSEA4 was observed from flow cytometric analysis (Fig. 3c), while flow cytometric analysis of 3D-hESCs from various generations showed the same results (Supplementary Fig. 4). Live-dead cell staining results showed that most cells were alive within the aggregates (Fig. 3d). Moreover, single cells dissociated from hESC spheres could develop into teratomas in SCID mice comprising the derivatives of all three germ layers, which included the cartilage (mesoderm), respiratory epithelium (endoderm), and brain tissue (ectoderm) (Fig. 3e). Another hESC line, H7, could be observed with similar results in the system (Supplementary Fig. 5). These results indicated the capacity to self-renew and to differentiate into all three germ layers in the 3D culture system.

### Derivation and characterization of mesenchymal stem cells (MSCs) from hESCs (hESC-MSCs)

**Fig. 4:**
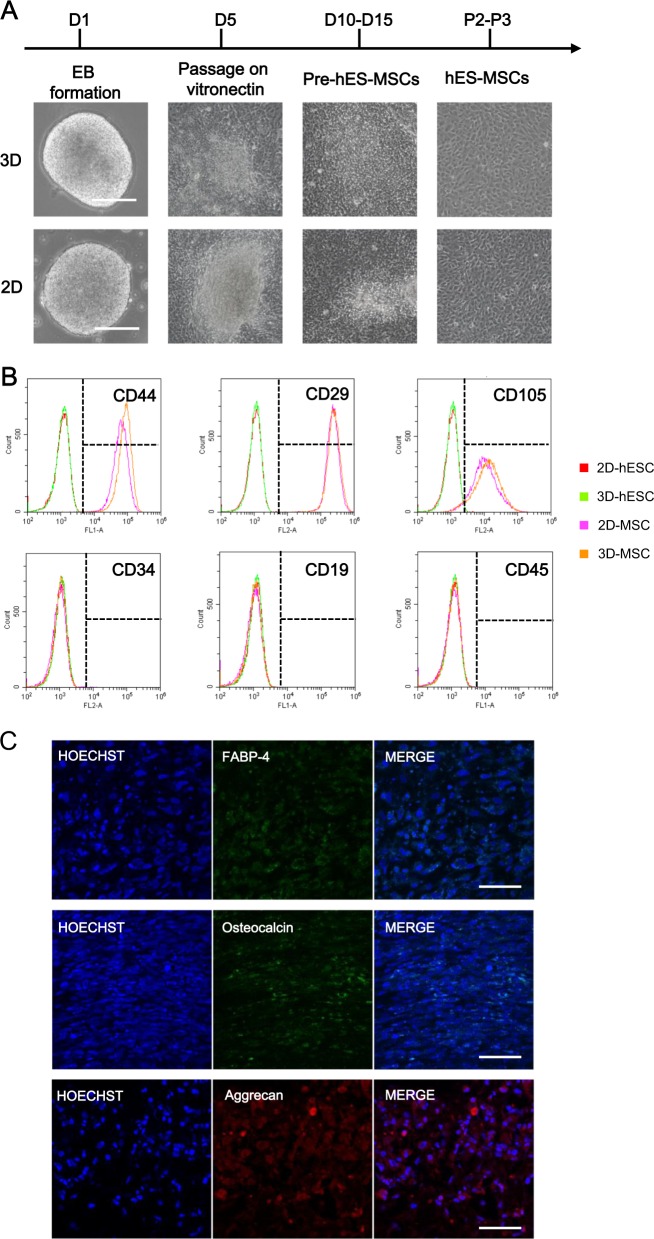
Comparison of MSCs derived from 2D-hESCs and 3D-hESCs. **a** The morphology of 2D- and 3D-hESC-MSCs at different stages of differentiation. **b** Flow cytometry analysis revealed specific MSC surface markers (CD44, CD29, and CD105) with negative controls (CD34, CD19, and CD45) in 2D- and 3D-hESC-MSCs. **c** Immunostaining of differentiated 3D-hESC-MSCs expressing an adipocyte marker (FABP-4), osteocytes maker (osteocalcin), and chondrocytes marker (aggrecan)

Consistent with being pluripotent, hESCs in 3D were used for directed in vitro differentiation. MSCs have been widely used in cell therapy clinical trials^37^. There are relatively few studies devoted to MSC differentiation from 3D-hESC spheres^38^. Here, we tried the differentiation using EB formation method (Fig. 4a, up)^39^. After 7-day suspension culture, EBs were attached to the plate and cultured in hESC-MSC derivation medium. Then cells were passaged at a 70–80% confluency. After 6 passages, both 2D- and 3D-hESC-MSCs exhibited similar morphology with typical MSCs (Fig. 4a). Surface markers of MSCs were analyzed, and maintained at high levels in the MSCs (Fig. 4b). Moreover, 3D-hESC-MSCs can be induced to differentiate into adipocytes, osteocytes, and chondrocytes (Fig. 4c). These results suggested that 3D-hESCs had the same ability of MSC differentiation as 2D-hESCs.

## Discussion

In this study, we have successfully developed a simple, economical, and robust static suspension culture system for large-scale hESC expansion yielding 1.5 × 10^9^ cells per 1.5-L system while maintaining normal characteristics of hESCs. To our knowledge, the hESC suspension culture system we established has been the largest static one so far and it is applicable in labs without employing expensive equipment. Compared with previous reports of others^40^, which showed hESC survival encapsulated in PNIPAAm-PEG hydrogel, our work showed hESC survival in liquid suspension culture system. Many studies have indicated 3D conditions influencing stem cell fates^41^. It is still unclear whether PNIPAAm-PEG would induce or contribute to PSC differentiation especially for neural differentiation because the elastic modulus of PNIPAAm-PEG (10%) is around 1000 pa, which is similar to the modulus of native neural tissues^42^ and might tend to initiate neural differentiation^43^. The data of Lei et al. have supported that PNIPAAm-PEG (8–10%) is softer than some other normal hydrogels, but the risky still exists. To support expansion for single-cell seeding, they treated the system with ROCK inhibitor, Y27632, for 4 days (96 h). However, long-term (over 96 h) addition of Y27632 may reduce the stemness levels of hPSCs^44^. Also, the prolonged exposure of Y27632 during the suspension culture of dissociated hPSCs promoted the inducing into retinal progenitors and telencephalic precursors^45,46^. Moreover, our system is much cheaper and simpler because it is not essential to refresh the media everyday and there is no expansive biomaterials or devices involved.

Clump formation is the main concern in static suspension cultures for causing cell apoptosis or unexpected differentiation^47^. We justified the optimal density of 2.0 × 10^5^ cells/ml, with which hESCs spontaneously formed round spheres with a uniform size of about 200 μm in diameter during culture (Supplementary Fig. 1A). When cultured in ultra-low dishes, spheres rarely formed big clumps, and, after being transferred to culture bags, 1% methylcellulose in the medium could prevent clump formation by increasing viscosity^19,24^. Subculturing is also an essential step for robust hESC expansion. Mechanical passage through filters can improve the uniformity of spheres, but will cause mechanical damages to hESCs^48^. Therefore, we chose Accutase to digest D5-spheres and D6-spheres into single cells in a gentle manner, achieving a cell viability of over 90% (Fig. 1d). Treatment of 10 μM Y27632 further promoted cell survival for the first 48 h after inoculation, which also contributed to the long-term expansion, maintaining pluripotent features^46^. hESCs were passaged 30 times in series in this manner, and maintained pluripotency and differentiation potential. During the whole process, hESC spheres grew in a xeno-free, fully defined E8 medium without the supplement of other matrices or microcarriers, which was compliant with GMP^14,49,50^.

So far, some progress has been made on 3D differentiation systems. For example, Henning et al.^51^ generated 40–50 million hPSC-CMs at over 80% purity from a 100-mL bioreactor. However, most 3D differentiation systems were small scale^52–54^. The system in the present study can not only support hESC expansion in the static platform, but also may be applied in dynamic bioreactor systems. In fact, we have already started expanding hESCs in a fully instrumented bioreactor (DASbox/eppendorf). Though the parameters need to be further adjusted and controlled, we have achieved preliminary success in massive hESC proliferation. Meanwhile, we are making attempts to establish differentiation platforms for various cell lineages in our lab^22^. In summary, the system to expand hESCs on a large scale represents a first important step and provides an unprecedented opportunity for regenerative medicine.

## Materials and methods

### hESC line and medium

A clinical-grade hESC line, Q-CTS-hESC-2, which was derived by our lab and had been reported^22^, was used to optimize and establish this 3D culture system. Q-CTS-hES-2 colonies were stably cultured in the vitronectin-supported adherent system before being transferred to suspension culture. We also applied the established suspension culture system to a classic hESC line, H7^2^ to compare the cell proliferation of hESC spheres suspended in different culture mediums, including embryonic body culture medium (EB, KODMEM supplemented with 20% KOSR, 1% nonessential amino acids, 2 mM l-glutamine, and 0.1 mM β-mercaptoethanol), conditioned mediums (CM, DMEM supplemented with 20% FBS, 1% nonessential amino acids, 0.1 mM β-mercaptoethanol co-cultured with human feeder cells, collected after 24 h), monkey embryonic culture medium (3:1, three parts of EB and one part of commercially available media PSGro (StemRD,YS000087, a fully defined, serum-and feeder-free medium)), and E8 medium (Gibco, A151700).

### Sphere culture of hESCs

To initiate suspension culture, hESC colonies in vitronectin-supported adherent cultures were dissociated into single cells by Accutase (Gibco, A11105-01). Digestion was terminated by adding an equivalent amount of E8 medium. We collected cell suspension, centrifuged and removed the supernatant, then resuspended hESCs with appropriate amount of E8 medium. After cell counting by Countess combined with trypan blue staining, we took a proper amount from the resuspension, and added it to the E8 medium supplemented with Y27632 (Selleck, S1049, final concentration was 10 μM), reaching a cell density of 2.0 × 10^5^ cells/mL. In total, 10 mL of cell resuspension was seeded into each 100-mm ultra-low dish (Corning, 3471), then placed in 37 °C incubators, and shaken well. The medium was changed 48 h after inoculation for sphere formation; afterwards, cultures were daily refreshed till D5, and then underwent passage. Sphere morphology was observed by microscopy. Cell counting and viability test were carried out every time after a subculturing.

### Scaling up to cell culture bags

For progressively scaling up, we employed cell culture bags (TaKaRa, FU005 & GT-T610A). At least 1.0 × 10^7^ hESCs (50-ml suspension) were needed for a 250-ml bag-based culture platform, and at least 6.0 × 10^7^ hESCs (300-ml suspension) were required for a 1.8-L bag-based system. Seeding density, passage method, and medium change were the same as for the dish-based culture system; moreover, 1% of methylcellulose (R&D, HSC001) was supplemented when the inoculation volume reached 100 ml in order to prevent clump formation. Sphere morphology was observed by microscopy. Cell counting and viability test were carried out every time after a subculturing.

### Characterization of hESCs in 3D

The diameters of the hESC spheres were measured and analyzed using photographs, which were taken by microscopy and processed with ImageJ software. Cells were counted with Countess and viability was examined by trypan blue staining. The fold increase in cell number was calculated using cell numbers on D0 and D5 or D6, according to the duration of the intervals. After hESCs stably proliferated in 3D systems for more than 10 passages, karyotype analysis and G-binding were conducted at the Chinese Academy of Medical Science & Peking Union Medical College.

For pluripotency detection, hESC spheres were immunostaining, flow cytometry analysis, and qRT-PCR. For immunostaining, hESC spheres were fixed with 4% paraformaldehyde, rinsed in PBS, sequentially dehydrated with 10%, 20%, and 30% sucrose, and embedded in optimum cutting temperature compound for frozen sectioning, and then samples were sectioned in 15-μm-thick slices. Slices were permeabilized by 1% Triton-X100, blocked with 2% BSA, and stained with primary antibodies as follows: goat anti-OCT3/4 (Santa Cruz Biotechnology, k0615) and mouse anti-NANOG (Santa Cruz Biotechnology, k2811). The samples were incubated with secondary antibodies followed by nuclear staining. Signals were detected and photographed through fluorescence microscopy.

For flow cytometry analysis, spheres were digested with Accutase for 3 min, dissociated into single cells by pipette, and then underwent fixation, permeation, and blocking as described in immunostaining. For indirect labeling, samples were stained with diluted primary antibody solution, followed by secondary antibody incubation. Data were collected on the flow cytometry and analyzed using FlowJo software. For direct labeling, samples were stained with direct-label antibodies, and then analyzed.

For marker gene expression analysis, total RNA was extracted using RNAprep pure Micro Kit (TIANGEN, DP420). cDNA was then synthesized using the extracted RNA and Prime Script^TM^ RT reagent kit. Quantitative real-time PCR (qRT-PCR) was performed as previously described. The details of the primers for hESCs were listed as follows: OCT4-s: GAGGAGTCCCAGGACATCAAAG, OCT4-a: CAGATGGTCGTTTGGCTGAATA, SOX2-s: ATGGCGAGCGGGGTTGG, SOX2-a: TCTGCGAGCTGGTCATGGAGTT. P53-a: CAGCACATGACGGAGGTTGT, P53-s: TCATCCAAATACTCCACACGC, MYC-a: GGCTCCTGGCAAAAGGTCA, MYC-s: CTGCGTAGTTGTGCTGATGT, GAPDH-s: AGGCATCCTCACCCTGAAGTA, GAPDH-a: CACACGCAGCTCATTGTAGA.

### Teratoma formation

For teratoma formation, D-5 hESC spheres were dissociated into single cells as described in sphere culture of hESCs, concentrated in PBS at a density of 5 × 10^7^ cells/ml. In total, 1.0 × 10^6^ cells were injected into each testis of 6-week-old CB17 SCID male mice under a sterile stereo microscope. Teratomas were isolated after 2 months of slice section and HE staining as described previously^22^.

### Generation and characterization of MSCs from 2D- and 3D-hESCs

Both 2D-hESC colonies and 3D-hESC spheres were digested into single cells and seeded in ultra-low attachment dish (Corning, 3471) for EB formation, respectively. hESC-EBs were cultured in EB medium (77% KODMEM + 20% KOSR + 1% NEAA + 0.1% β-mercaptoethanol + 1% glutamax + 1% penicillin/streptomycin) for 4 days and then passaged on vitronectin. hMSC differentiation medium (82% KODMEM + 15% FBS + 1% NEAA + 0.1% β-mercaptoethanol + 1% glutamax + 1% penicillin/streptomycin + 10 ng/ml bFGF + 5 ng/ml TGFb)^55^ was employed and the medium was changed every other day. Pre-hESC-MSCs could be observed from D12 to D15, and hESC-MSCs were harvested after separation and expansion.

For flow cytometry analysis, hESC-MSCs were digested with Tryple (Gibco, A1285901) for 3 min, and then underwent fixation, permeation, and blocking as described before. For direct labeling, MSCs were stained with FITC anti-CD44 (BD, 555478), PE anti-CD29 (Biolegend, 303004), PE anti-CD105 (Biolegend, 323206), PE anti-CD34 (BD, 555822), FITC anti-CD19 (BD, 555412), FITC anti-CD45 (eBioscience, 11-9459-42), and analyzed using FlowJo software.

For adipogenic, osteogenic, and chondrogenic differentiation, hESC-MSCs were digested by Tryple, resuspended by fresh MSC culture medium (MesenCult™ MSC Basal Medium + MesenCult™ MSC Stimulatory Supplement, STEMCELL Technologies), and seeded into four-well plates. MSC culture medium was then replaced by Human Mesenchymal Stem Cell Functional Identification Kit (R&D, SC006, prepared as instruction manual told). For adipogenic differentiation, fat vesicles could be observed 1–3 weeks later. For osteogenic differentiation, calcium deposition could be observed 3 weeks later. Immunofluorescence staining was performed as previously described. Samples were subsequently incubated with primary antibodies against FABP (Pierce, PA5-30591, 1:200), osteocalcin (Pierce, PA5-11849, 1:200), and aggrecan (Pierce, MA3-16888, 1:200) at 4°C overnight, and then incubated with secondary antibodies.

### Statistical analysis

Statistical analyses were performed in IBM SPSS Statistics 22. All results were expressed as the mean ± SD. The unpaired two-tailed Student’s *t*-test was used to compare the mean values of measurements. Differences were considered significant for *p* < 0.05.

## Electronic supplementary material

Supplemental Figures

supplementary figure legends
