# Supplementary material for: A fully defined static suspension culture system for large-scale human embryonic stem cell production
Source: Cell Death Dis. 2018 Aug 30;9(9):892. doi: 10.1038/s41419-018-0863-8 (PMC6117302; doi:10.1038/s41419-018-0863-8)
Supplement: Supplementary file 1 — Supplemental Figures [file 41419_2018_863_MOESM1_ESM.pptx]

## Slide 1
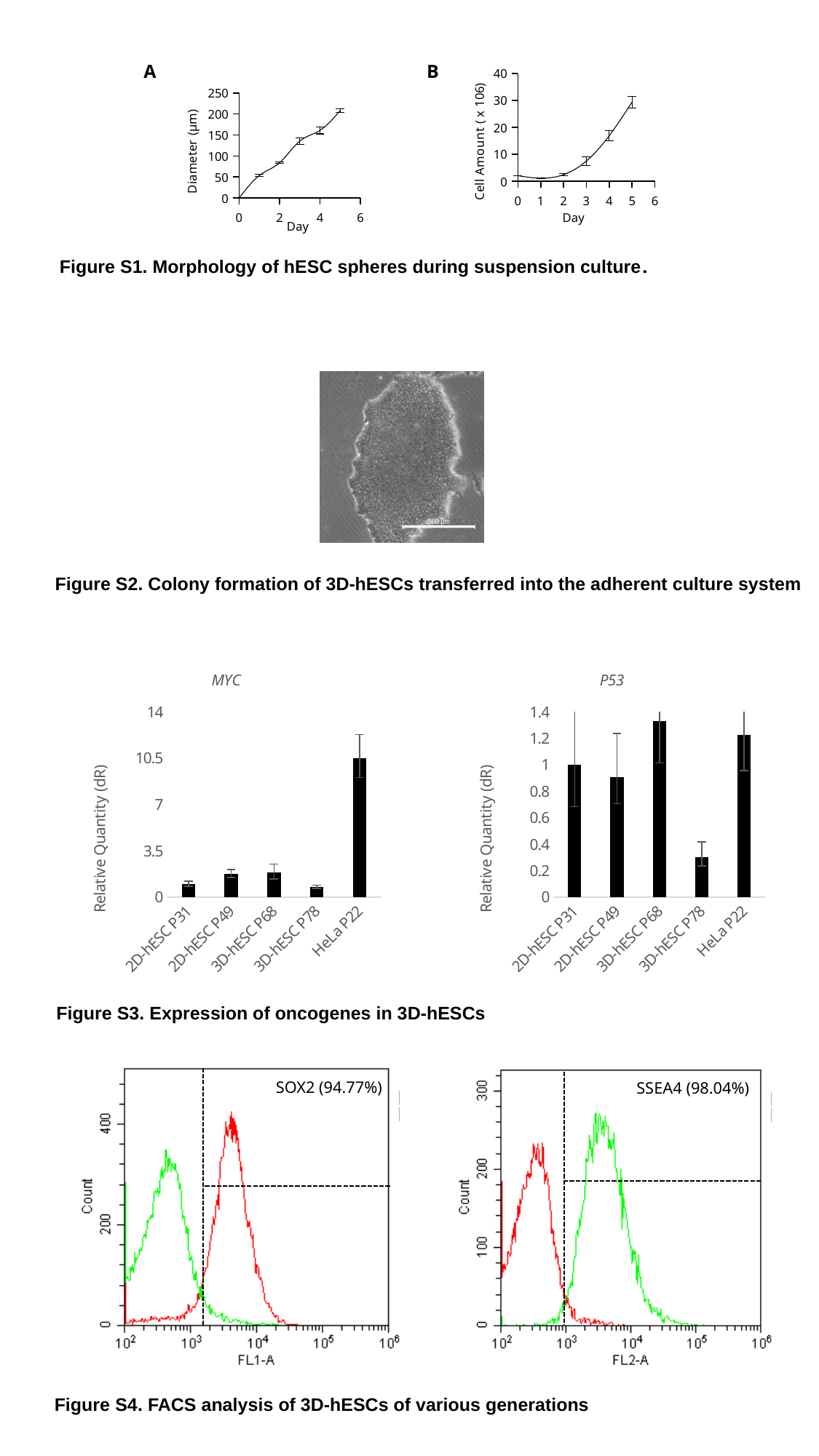

A
B
### Chart
| Category | |
|---|---|
### Chart
| Category | |
|---|---|Figure S1. Morphology of hESC spheres during suspension culture.
Figure S2. Colony formation of 3D-hESCs transferred into the adherent culture system
### Chart: MYC
| Category | MYC |
|---|---|
| 2D-hESC P31 | 1.0 |
| 2D-hESC P49 | 1.74978 |
| 3D-hESC P68 | 1.85681 |
| 3D-hESC P78 | 0.76338 |
| HeLa P22 | 10.51685 |
### Chart: P53
| Category | P53 |
|---|---|
| 2D-hESC P31 | 1.0 |
| 2D-hESC P49 | 0.90858 |
| 3D-hESC P68 | 1.33061 |
| 3D-hESC P78 | 0.30417 |
| HeLa P22 | 1.2258 |Figure S3. Expression of oncogenes in 3D-hESCs
SOX2 (94.77%)
SSEA4 (98.04%)
Figure S4. FACS analysis of 3D-hESCs of various generations

## Slide 2
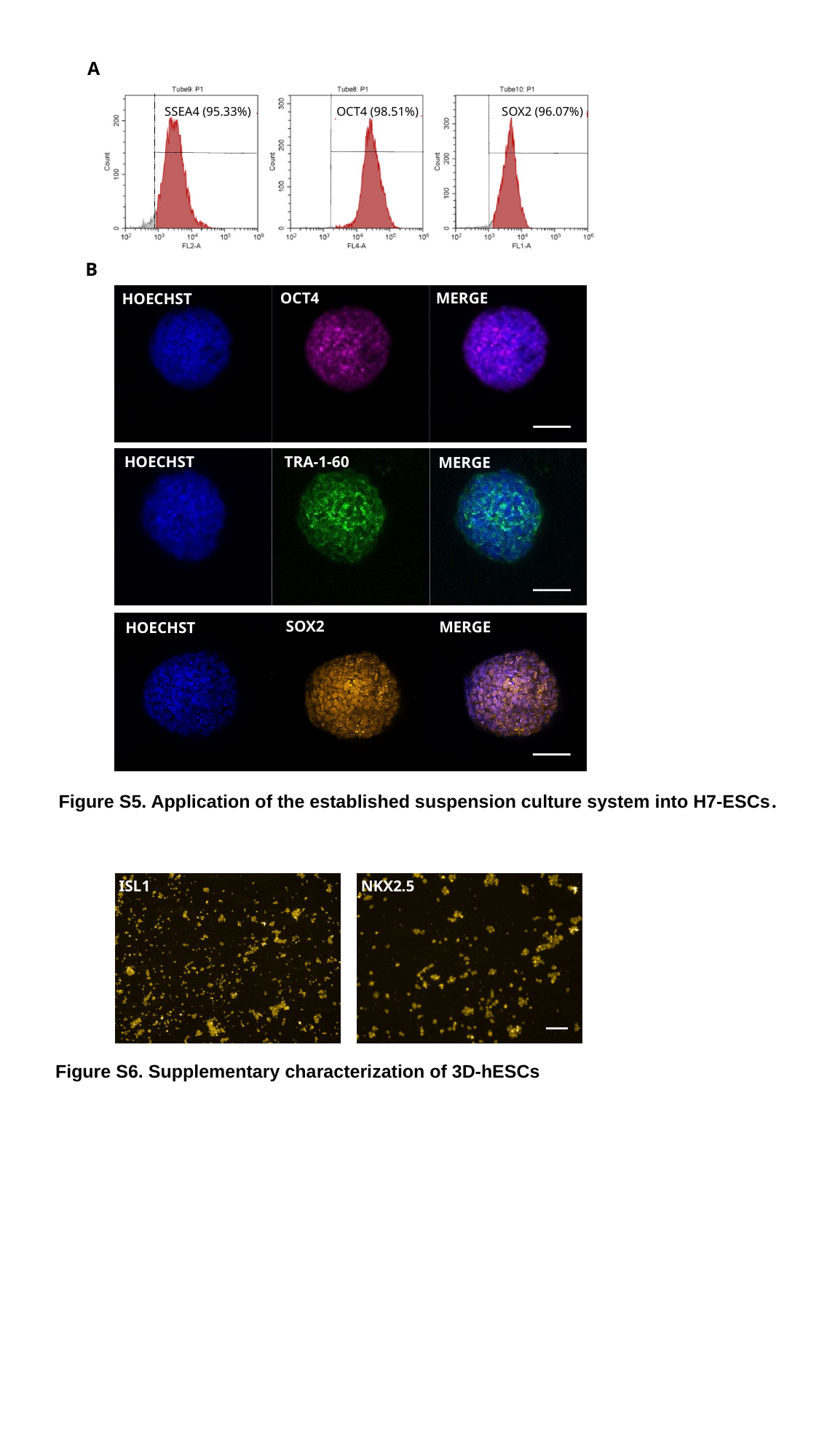

A
SSEA4 (95.33%)
OCT4 (98.51%)
SOX2 (96.07%)
B
OCT4
MERGE
HOECHST
TRA-1-60
HOECHST
MERGE
SOX2
MERGE
HOECHST
Figure S5. Application of the established suspension culture system into H7-ESCs.
ISL1
NKX2.5
Figure S6. Supplementary characterization of 3D-hESCs
