# Supplementary material for: A fully defined static suspension culture system for large-scale human embryonic stem cell production
Source: Cell Death Dis. 2018 Aug 30;9(9):892. doi: 10.1038/s41419-018-0863-8 (PMC6117302; doi:10.1038/s41419-018-0863-8)
Supplement: Supplementary file 2 — supplementary figure legends [file 41419_2018_863_MOESM2_ESM.docx]

**Figure S1. Morphology of hESC spheres during suspension culture.**

1. Growth curve of hESCs expanded in an interval. (B) Diameter curve of hESC spheres in an interval.

**Figure S2. Colony formation of 3D-hESCs transferred into the adherent culture system**

3D-hESCs spheres formed normal colonies like 2D hESCs with stable passages. Scale bars, 500 μm.

**Figure S3. Expression of oncogene s in 3D-hESCs**

Comparative oncogene expression between Hela, 2D- and 3D-hESCs. Relative gene expression represents data normalized to *GADPH* and expressed relative to 2D-hESC with P31 (2D-hESC P31). Mean ± S.D.

**Figure S4. FACS analysis of 3D-hESCs of various generations**

Flow cytometry of 3D-hESCs of various generations expressing SOX2 and SSEA4.

**Figure S5. Application of the established suspension culture system into H7-ESCs**

(A) FACS analysis revealed positive expression ratio of over 95% for all of the examined pluripotent markers. (B) Immunostaining of hESC spheres for H7 cell line.
